# Evaluation of and Intervention for Sarcopenia in Hepatology Departments: A Survey of Nurses in Japan

**DOI:** 10.3390/healthcare9081021

**Published:** 2021-08-09

**Authors:** Kazuki Ohashi, Madoka Ito, Megumi Kawakubo, Ikue Sato

**Affiliations:** 1 Sapporo University of Health Sciences, Sapporo 007-0894, Japan; m-ito@sapporo-hokeniryou-u.ac.jp (M.I.); ikue_sato@sapporo-hokeniryou-u.ac.jp (I.S.); 2 Saga Medical School, Saga 840-8501, Japan; sh2335@cc.saga-u.ac.jp

**Keywords:** sarcopenia, clinical practice, chronic liver disease, evaluation

## Abstract

Sarcopenia is associated with poor prognosis and decreased quality of life in patients with chronic liver disease (CLD). The present study aimed to clarify the dissemination of interventions such as evaluations, prevention efforts, and treatments for sarcopenia among patients in hepatology outpatient departments and wards in Japan, as well as examine the factors related to such dissemination. A cross-sectional study was performed involving nurses from hospitals accredited by the Japan Society of Hepatology. Participants completed a questionnaire regarding evaluations and interventions for sarcopenia in their department. Nurses from 72 outpatient departments and 162 wards provided responses to the questionnaire. Overall, 37.9% of outpatient departments and 37.6% of wards performed evaluations or interventions for sarcopenia. Outpatient departments and wards that evaluated sarcopenia or intervened held more workshops or training regarding sarcopenia than departments and wards that did not (outpatient departments: 52.0% vs. 12.2%, wards: 32.1% vs. 12.9%). Holding workshops or training regarding sarcopenia (outpatient departments; OR = 7.51, 95% confidence interval (CI): 2.12–26.6, wards; OR = 2.61, 95% CI: 1.11–6.15) was significantly associated with dissemination practices. These findings suggest that expanding knowledge of sarcopenia and developing practical skills among general nurses may aid in preventing sarcopenia among patients with CLD.

## 1. Introduction

Sarcopenia is a muscle disease characterized by loss of muscle strength and mass accompanied by a decline in physical function [1,2]. Several studies have reported that sarcopenia is an important factor influencing clinical outcomes in patients with various diseases [3]. In general, the prevalence of sarcopenia in Japanese older adults is 11.6% in men and 16.7% in women [4]. In contrast, the prevalence of sarcopenia in patients with liver cirrhosis (LC) or hepatocellular carcinoma (HCC) is higher than that in healthy older adults [5]. A 2017 survey estimated that approximately 75% of patients with chronic liver disease (CLD) were over 65 years of age [6]. Hence, many patients with CLD in Japan have a high risk of sarcopenia. Moreover, sarcopenia is associated with increased complications after hepatectomy and liver transplantation and with poor prognosis in patients with LC [7,8]. Thus, early detection, prevention, and treatment of sarcopenia are important issues in the clinical practice of hepatology.

In Japan, the criteria described by the European Working Group on Sarcopenia in Older People 2 (EWGSOP2) [2], the Asian Working Group for Sarcopenia (AWGS) [3], and the Japan Society of Hepatology (JSH) [5] are commonly used to diagnose sarcopenia in patients with CLD. According to these criteria, hand grip strength (HGS) is recommended for routine evaluation of sarcopenia in clinical practice and community healthcare because of its ease of use [2]. Similarly, body impedance analysis (BIA) is a simple and non-invasive method for estimating muscle mass. The SARC-F is a recently developed questionnaire that makes it possible to identify sarcopenia without special equipment in outpatient departments, at the patient’s bedside, and in the community [9]. These developments have increased the opportunities for nurses to intervene in cases of sarcopenia.

Nurses who provide care that focuses on the patients’ daily lives can play a valuable role in interventions for sarcopenia [10]. Nurses can contribute to the prevention of sarcopenia by managing the nutritional status and dietary and exercise habits of patients. A recent study on the treatment of patients with CLD accompanied by sarcopenia has demonstrated that nutritional prescription and exercise can be used to effectively manage sarcopenia and liver disease [11]. Other studies have reported that increasing the number of daily steps and administering branched-chain amino acids improves HGS and leg strength [12], and that exercise during hospitalization prevents loss of skeletal muscle mass [13].

A previous study demonstrated that hospitalization causes sarcopenia in approximately 15% of patients, even if sarcopenia is not reported before admission [14]. Acute disease and trauma, as well as inappropriate management of nutritional care by medical staff and patient inactivity, are among the causes of iatrogenic sarcopenia during hospitalization. The behavior of medical professionals may trigger iatrogenic sarcopenia in healthcare facilities [15,16]. Therefore, nurses should collaborate with other healthcare professionals to evaluate sarcopenia, manage nutrition, and reduce inactivity among patients.

Several studies have examined the knowledge or awareness of sarcopenia among nurses [17,18]. A survey of nurses belonging to the Japanese Association of Rehabilitation Nutrition reported that less than half of them evaluated muscle strength (49.1%), muscle mass (45.3%), and physical function (47.2%) in their patients [17]. In addition, half of the nurses who were surveyed in Australia and New Zealand did not think that diagnosing sarcopenia was part of their role [18]. Thus, half of nurses may be uninterested or take no action about sarcopenia. The differences in knowledge and awareness among healthcare professionals represent barriers to the diagnosis and treatment of sarcopenia.

Despite evidence that it leads to poor prognosis, knowledge of sarcopenia appears to be uncommon among nurses. A previous study revealed that patients with LC with sarcopenia exhibit a mortality rate 3.23 times higher than that of their counterparts without sarcopenia [8]. Thus, nurses nationwide should intervene to improve health and quality of life among patients with sarcopenia. However, few studies have evaluated the dissemination of interventions in hepatology outpatient departments and wards.

Therefore, the purpose of this study was to clarify the dissemination of interventions such as evaluations, prevention efforts, and treatments for sarcopenia in hepatology departments and wards, as well as examine the factors related to such dissemination. Our findings indicated that holding workshops or training regarding sarcopenia was significantly associated with dissemination practices, suggesting that expanding knowledge of sarcopenia and developing practical skills among general nurses can aid in preventing sarcopenia among patients with CLD.

## 2. Materials and Methods

### 2.1. Study Design

This cross-sectional study included nurses who belonged to hospitals accredited by the JSH. As of 2019, there were 474 accredited hospitals in Japan. The conditions for accredited hospitals that were included were as follows: (1) there were 10 or more beds for hepatology, (2) at least three board-certified hepatologists of the JSH were enrolled, and at least one of them was a fellow. Specifically, accredited hospitals routinely provide care to patients with CLD. This study focused on hepatology departments in the hospital. The questionnaire regarding interventions in the department was sent to the hepatology outpatient department and hepatology ward at each hospital, and one representative nurse who manages the department completed the questionnaire. The survey was administered between 1 October 2020, and 31 December 2020.

### 2.2. Data Collection

The questions were as follows: location (prefecture), hospital type, number of beds, internal medicine or surgery, enrollment of certified nurses (CN), enrollment of certified nurse specialists (CNS), enrollment of a hepatitis medical care coordinator, utilization of workshops or training regarding sarcopenia, presence of an organized nutrition support team, use of criteria for diagnosing sarcopenia, evaluation items related to sarcopenia, intervention for sarcopenia, open-ended questions regarding interventions for sarcopenia, enrollment of a ward-specific physiotherapist, and enrollment of a ward-specific dietician. Previous studies and experts have hypothesized that these items contribute to the implementation of evaluations and interventions for sarcopenia [10,11,17].

### 2.3. Ethical Considerations

Written informed consent was obtained from all participants. Personal information and personal opinions were not collected. Therefore, it was not possible to identify an individual after the participants returned the questionnaire. This study was approved by the ethics committee of Sapporo University of Health Sciences (approval number: 020002-4).

### 2.4. Statistics Analyses

Data for the outpatient departments and wards were analyzed separately. The departments that evaluated sarcopenia or sarcopenia interventions were defined as the practice group (PG), and those that did not were defined as the non-practice group (NPG). Nominal and categorical variables were expressed as numbers (%) and compared using the Fisher’s exact test. First, we clarified the characteristics of the PG and NPG. Second, logistic regression analysis was used to analyze the factors associated with sarcopenia interventions. We identified factors associated with the PG using logistic regression analysis, which was performed using a set of covariates that were likely to be related to implementation in the outpatient department. These covariates included hospital size (≥400 beds), enrollment of a CN or CNS, and utilization of workshops or training regarding sarcopenia. In the ward, the covariates included hospital size (≥400 beds), enrollment of a CN or CNS, utilization of workshops or training regarding sarcopenia, enrollment of a ward-specific physiotherapist, and enrollment of a ward-specific dietician. Finally, for subgroup analysis, we examined the characteristics of departments that evaluated sarcopenia or intervened in patients with sarcopenia. All analyses were performed using EZR version 1.54 (Jichi Medical University, Japan) [19]. Statistical significance was set at *p* < 0.05.

## 3. Results

Overall, nurses from 72 (15.2%) outpatient departments and 162 (34.2%) wards answered the questionnaire. The questionnaires with missing values were eliminated (6 outpatient departments and 13 wards). Accordingly, the data obtained from 66 outpatient departments (valid response rate, 91.7%) and 149 wards (valid response rate, 92.0%) were analyzed (Table 1).

Furthermore, 37.9% (*n* = 25) of outpatient departments and 37.6% (*n* = 56) of wards performed evaluations or interventions for sarcopenia. Among outpatient departments, the PG was characterized by greater utilization of workshops or trainings regarding sarcopenia than the NPG (52.0% vs. 12.2%). Although use of sarcopenia diagnostic criteria was significantly higher in the PG than in the NPG, only 24% of the PG used the criteria. Among hepatology wards, the PG also held more workshops or training regarding sarcopenia than the NPG (32.1% vs. 12.9%), and 64.3% of wards in the PG used the diagnostic criteria for sarcopenia. Although not significant, enrollment of a ward-specific physiotherapist was lower in the PG than in the NPG (48.2% vs. 64.5%, *p* = 0.06) (Table 2). Logistic regression analysis indicated that holding workshops or training regarding sarcopenia (outpatient department; OR = 7.51, 95% CI: 2.12–26.60, ward; OR = 2.61, 95% CI: 1.11–6.15) was significantly associated with the dissemination of sarcopenia interventions (Table 3).

For the subgroup analysis, we compared findings for the groups that did (EG) and did not (NEG) perform evaluations for sarcopenia (Table 4). The EG accounted for 27.3% of outpatient departments (*n* = 18). Additionally, the use of diagnostic criteria was significantly higher in the EG than in the NEG (33.3% vs. 2.1%). In contrast, for wards, utilization of workshops or trainings regarding sarcopenia (32.6% vs. 15.1%), use of diagnostic criteria, and prevention or treatment of sarcopenia were more frequent in the EG than in the NEG.

**Figure 1:**
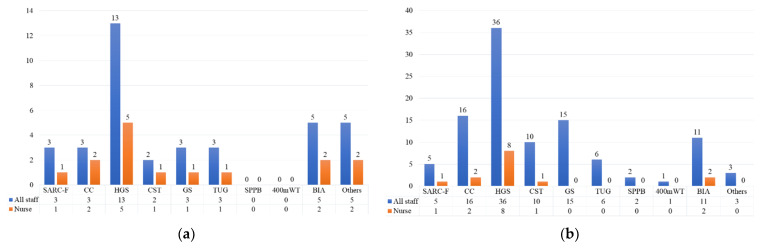
Items related to the evaluation of sarcopenia in outpatient departments (multiple choice questions). (**a**) Outpatient departments. (**b**) Wards. CC, calf circumference; HGS, hand grip strength; CST, chair stand test; GS, gait speed; TUG, timed up-and-go test; SPPB, Short Physical Performance Battery; 400 mWT, 400-m walk test; BIA, bioelectrical impedance analysis; Others.

The rate of enrollment of ward-specific physiotherapists was lower in the EG than in the NEG (41.9% vs. 65.1%). The evaluation items for sarcopenia are described in Figure 1. HGS was the most common measure used in both outpatient departments and wards and by nurses.

Lastly, we compared findings between two groups categorized based on the utilization of sarcopenia interventions (including prevention) (IG, intervention group) or lack thereof (NIG, non-intervention group) (Table 5). Among outpatient departments, utilization of workshops or training regarding sarcopenia was higher in the IG than in the NIG (84.6% vs. 13.2%). Among wards, utilization of workshops or training regarding sarcopenia (41.4% vs. 15.0%) and the use of diagnostic criteria (44.8% vs. 7.5%) were more common in the IG group than in the NIG group. Details related to prevention or treatment that were obtained from open-ended questions are included in Table 6. Most of the interventions involved nutritional and exercise therapy. These interventions were administered through education, guidance, or direct treatment.

## 4. Discussion

The current study investigated the dissemination of interventions for sarcopenia in patients with CLD in Japan. To the best of our knowledge, this is the first study to perform such an evaluation. Our findings indicated that approximately 40% of hepatology outpatient departments and wards performed evaluations or interventions for sarcopenia. In addition, holding workshops or training regarding sarcopenia was a significant factor influencing the introduction of sarcopenia evaluations and interventions, suggesting that lack of knowledge regarding sarcopenia contributes to decreased utilization of prevention, evaluation, and intervention strategies. Thirty years have passed since sarcopenia was described by Rosenberg [20]. In 2016, sarcopenia was registered in the ICD-10 by the World Health Organization [21]. In Japan, clinical guidelines for sarcopenia were published in 2017 [22,23,24,25]. According to the study by Nakahara et al. [17], there are differences in the practice of sarcopenia assessment among healthcare professionals, and sarcopenia has only recently been defined as a disease and a treatment target. The knowledge and skills that are required to manage sarcopenia are expanding. The association between holding workshops or training regarding sarcopenia and the practice of evaluating and intervening in patients with sarcopenia is consistent with the current clinical situation.

Approximately 30% of outpatient departments and wards evaluated sarcopenia. HGS measurements were the most common assessments in both departments and wards. Nurses were also involved in HGS measurements. HGS is the most significant parameter for diagnosing sarcopenia according to the general criteria [2,3,5]. Moreover, studies have indicated that HGS can be an independent predictor of composite hepatic events, such as hepatic decompensation, ascites, and hepatic encephalopathy [26]. Therefore, our results that HGS is more common than other assessments are reasonable. Previous studies have revealed that low HGS is related to complications after surgery and the length of hospital stay in older adults [27,28], and that low HGS on admission is a predictor of low physical function at discharge [29]. Moreover, men with HGS >31 kg and women with HGS >18 kg upon admission were more likely to be able to return home from the hospital [30]. Ibrahim et al. reported that implementing HGS as a routine measurement upon admission was more feasible [31]. Because of its simplicity and low cost, it can be easily understood and implemented by many staff members. For nurses, it is easy to obtain HGS measurements when collecting other routine anthropometric data such as height and weight. Measuring HGS may provide an early opportunity to identify patients with CLD likely to exhibit poor prognoses, which may aid in selecting appropriate strategies for improving prognosis.

About 40% of outpatient departments and wards intervened to prevent or reverse sarcopenia. Exercise and nutritional management were central to these interventions. Previous studies have demonstrated that the administration of leucine prevents sarcopenia in patients with LC [32]. For patients with LC, a combination of aerobic and resistance exercise has been recommended after managing the frequency, intensity, type, and duration of exercise [11]. Healthcare professionals may be able to prevent inpatient sarcopenia through direct treatment. However, outpatients must be provided with knowledge regarding sarcopenia and skills that can motivate them to improve their dietary and exercise habits. Based on responses to the open-ended questions regarding sarcopenia interventions, a combination of patient education and direct treatment is often implemented. Since the current guidelines [24,25] identify treatment strategies for sarcopenia, implementation is expected in many facilities.

Recently, “sarcopenia” has been incorporated into the curriculum of nursing education by the Ministry of Education, Culture, Sports, Science and Technology [33]. Understanding the concepts of frailty, sarcopenia, and locomotive syndrome and practicing preventive nursing is among the key learning goals of nursing education. This concept involves learning nursing practices for health promotion and disease prevention. For nurses, knowledge and practical skills are required to address issues affecting the aging population in Japan. However, awareness of sarcopenia among nurses may be limited to primary health care. EWGSOP2 has identified the subcategories of sarcopenia as acute and chronic. Sarcopenia lasting <6 months is considered an acute condition, while sarcopenia lasting ≥6 months is considered a chronic condition [2]. This means that sarcopenia must be evaluated and addressed at every stage. Sarcopenia can be managed by a variety of healthcare professionals. Especially, rehabilitation nutrition maximizes performance through the effective collaboration of healthcare professionals [16]. However, in our study, enrollment of a ward-specific physiotherapist tended to be a negative factor in practice (Table 2). Because this study was based on nurses’ responses to a questionnaire, evaluations and interventions performed by nurses and physiotherapists may have differed. Nurses can contribute to the medical team’s handling of sarcopenia by establishing knowledge and practical skills using nursing education programs.

### Strengths and Limitations

Our study clarified the evaluations and interventions for sarcopenia implemented among patients in hepatology outpatient departments and wards. Moreover, our findings highlight the importance of expanding knowledge and skills related to sarcopenia among both patients and medical professionals. The results suggest that evaluations and interventions for sarcopenia can be expanded at a low cost regardless of hospital size or human resources. However, our study also has several limitations. First, the participants were representative of nurses in the department. Although the nurse who manages the department (e.g., the head nurse) was asked to complete the questionnaire, it is possible that the perceptions of healthcare professionals may have been ignored and that the participants may have reported incorrect information about the facility. Second, this study may have been influenced by selection bias. In other words, the participants who belonged to facilities that did not perform sarcopenia evaluations or interventions may have avoided answering the questionnaire. Thus, it is possible that rates of sarcopenia evaluation and intervention were lower than those indicated by our results. Third, outpatient departments and wards in this study may have included patients with non-liver diseases. Although the questionnaire was sent to departments dealing with liver diseases, we did not require them to be exclusive or specific to such diseases. Therefore, medical care for other diseases may have influenced the results of this study. Fourth, we did not consider the enrollment of pharmacists. A previous study revealed that polypharmacy was a risk factor for sarcopenia [34]. Finally, this study did not collect data on the proportion of patients aged over 65 years in the facilities. Basically, sarcopenia is an age-related disease. Thus, sarcopenia is likely a familiar issue for healthcare professionals who often meet elderly patients.

## 5. Conclusions

Despite the limitations of this study, our findings provide insight into the dissemination of sarcopenia evaluations and interventions in patients with CLD. Factors related to the utilization of such practices included holding workshops or training regarding sarcopenia in the hospital. Therefore, expanding knowledge of sarcopenia and developing practical skills among general nurses may aid in preventing sarcopenia among patients with CLD.

## Figures and Tables

**Table 1:** Question items and sample characteristics.

Items	Outpatient Department (*n* = 66) Number, (%)	Ward (*n* = 149) Number, (%)
Location		
Hokkaido	4 (6.1)	7 (4.7)
Tohoku	2 (3.0)	10 (6.7)
Kanto	12 (18.2)	31 (20.8)
Chubu	20 (30.3)	24 (16.1)
Kiniki	15 (22.7)	43 (28.9)
Chugoku	5 (7.6)	10 (6.7)
Shikoku	1 (1.5)	3 (2.0)
Kyushu-Okinawa	7 (10.6)	21 (14.1)
Hospital type		
National	7 (10.6)	14 (9.5)
Public medical	28 (42.4)	63 (42.9)
Social insurance	4 (6.1)	3 (2.0)
Healthcare corporation	12 (18.2)	34 (23.1)
Private clinic and others	15 (22.7)	33 (22.4)
No response		2
Number of Beds		
Beds < 400	26 (39.4)	42 (28.2)
Beds ≥ 400	40 (60.6)	107 (71.8)
Internal medicine or Surgery		
Internal medicine	44 (66.7)	100 (67.1)
Surgery	5 (7.6)	16 (10.7)
Mixed	17 (25.8)	33 (22.1)
Enrollment of CN or CNS		
Yes	45 (68.2)	61 (40.9)
No	21 (31.8)	88 (59.1)
Enrollment of the hepatitis medical care coordinator		
Yes	30 (45.5)	44 (29.5)
No	36 (54.5)	105 (70.5)
Holding workshops or training about sarcopenia.		
Yes	18 (27.3)	30 (20.1)
No	48 (72.7)	119 (79.9)
Nutrition support team		
Yes	64 (97.0)	149 (100.0)
No	2 (3.0)	0
Use of sarcopenia diagnostic criteria		
Yes	7 (10.6)	22(14.8)
No	59 (89.4)	127 (85.2)
Evaluation items related sarcopenia [in all staff]		
Yes	18 (27.3)	43 (28.9)
None	48 (72.7)	106 (71.1)
Evaluation items related sarcopenia [in nurse]		
Yes	13 (19.7)	12 (8.1)
None	53 (80.3)	137 (91.9)
Intervention for sarcopenia		
Yes	13 (19.4)	29 (19.5)
No	53 (80.3)	120 (80.5)
Enrollment of the ward-specific physiotherapist		
Yes		87 (58.4)
No		62 (41.6)
Enrollment of the ward-specific dietitian		
Yes		115 (77.2)
No		34 (22.8)

CN, certified nurse; CNS, certified nurse specialist. Location was expressed by grouping prefectures into regions.

**Table 2:** Comparison between practice group and non-practice group in outpatient department and ward.

	Outpatient Department			Ward		
	PG (*n* = 25)	NPG (*n* = 41)	*p*-Value	PG (*n* = 56)	NPG (*n* = 93)	*p*-Value
**Location**						
**Hokkaido**	2 (8.0)	2 (4.9)	0.16	1 (1.8)	6 (6.5)	0.64
**Tohoku**	1 (4.0)	1 (2.4)		5 (8.9)	5 (5.4)	
**Kanto**	4 (16.0)	8 (19.5)		9 (16.1)	22 (23.7)	
**Chubu**	5 (20.0)	15 (36.6)		10 (17.9)	14 (15.1)	
**Kinki**	9 (36.0)	6 (14.6)		16 (28.6)	27 (29.0)	
**Chugoku**	0	5 (12.2)		5 (8.9)	5 (5.4)	
**Shikoku**	0	1 (2.4)		2 (3.6)	1 (1.1)	
**Kyushu-Okinawa**	4 (16.0)	3 (7.3)		8 (14.3)	13 (14.0)	
**Hospital type**						
**National hospital**	2 (8.0)	5 (12.2)	0.92	5 (9.1)	9 (9.8)	0.3
**Public medical hospital**	12 (48.0)	16 (39.0)		24 (43.6)	39 (42.4)	
**Social insurance hospital**	1 (4.0)	3 (7.3)		3 (5.5)	0 (0.0)	
**Healthcare corporation**	5 (20.0)	7 (17.1)		11 (20.0)	23 (25.0)	
**Private clinic and others**	5 (20.0)	10 (24.4)		12 (21.8)	21 (22.8)	
**No response**				1	1	
**Number of Beds**						
**Beds < 400**	8 (32.0)	18 (43.9)	0.44	12 (21.4)	30 (32.3)	0.19
**Beds ≥ 400**	17 (68.0)	23 (56.1)		44 (78.6)	63 (67.7)	
**Internal medicine or Surgery**						
**Internal medicine**	18 (72.0)	26 (63.4)	0.78	35 (62.5)	65 (69.9)	0.68
**Surgery**	1 (4.0)	4 (9.8)		7 (12.5)	9 (9.7)	
**Mixed**	6 (24.0)	11 (26.8)		14 (25.0)	19 (20.4)	
**Enrollment of CN or CNS**						
**Yes**	20 (80.0)	25 (61.0)	0.17	28 (50.0)	33 (35.5)	0.09
**No**	5 (20.0)	16 (39.0)		28 (50.0	60 (64.5)	
**Enrollment of the hepatitis medical care coordinator**						
**Yes**	11 (44.0)	19 (46.3)	>0.99	19 (33.9)	25 (26.9)	0.46
**No**	14 (56.0)	22 (53.7)		37 (66.1)	68 (73.1)	
**Workshops or trainings about sarcopenia**						
**Yes**	13 (52.0)	5 (12.2)	<0.01	18 (32.1)	12 (12.9)	< 0.01
**No**	12 (48.0)	36 (87.8)		38 (67.9)	81 (87.1)	
**Nutrition support team**						
**Yes**	24	40	>0.99	56	93	
**No**	1	1		0	0	
**Use of sarcopenia diagnostic criteria**						
**Yes**	6 (24.0)	0	0.01	36 (64.3)	1 (1.1)	< 0.01
**No**	19 (76.0)	41 (100.0)		20 (35.7)	92 (98.9)	
**Enrollment of the ward-specific physiotherapist**						
**Yes**				27 (48.2)	60 (64.5)	0.06
**No**				29 (51.8)	33 (35.5)	
**Enrollment of the ward-specific dietitian**						
**Yes**				44 (76.3)	71 (76.3)	0.84
**No**				12 (21.4)	22 (23.7)	

Results are presented as number (%). Location was expressed by grouping prefectures into regions. PG, practice group; NPG, non-practice group; CN, certified nurse; CNS, certified nurse specialist.

**Table 3:** Multivariate logistic regression analysis for implementation.

Outpatient Department	OR	95% CI	*p*-Value
**Hospital size (400 ≤ beds = 1, beds < 400 = 0)**	0.97	0.15–0.66	0.96
**Enrollment of CN or CNS (yes = 1, no = 0)**	2.32	0.64–8.51	0.20
**Workshops or trainings about sarcopenia (yes = 1, no = 0)**	7.51	2.12–26.60	<0.01
**Ward**			
**Hospital size (400 ≤ beds = 1, beds < 400 = 0)**	1.65	0.73–3.69	0.23
**Enrollment of CN or CNS (yes = 1, no = 0)**	1.96	0.96–4.00	0.07
**Workshops or trainings about sarcopenia (yes = 1, no = 0)**	2.61	1.11–6.15	0.03
**Enrollment of the ward-specific physiotherapist (yes = 1, no = 0)**	0.45	0.20–1.01	0.05
**Enrollment of the ward-specific dietician (yes = 1, no = 0)**	1.73	0.66–4.50	0.26

OR, odds ratio; CI, confidential interval; CN, certified nurse; CNS, certified nurse specialist.

**Table 4:** Comparison between evaluate group and non-evaluate group in outpatient department and ward.

	Outpatient Department			Ward		
	EG (*n* = 18)	NEG (*n* = 48)	*p*-Value	EG (*n* = 43)	NEG (*n* = 106)	*p*-Value
**Location**						
**Hokkaido**	0	4 (8.3)	<0.01	1 (2.3)	6 (5.7)	0.49
**Tohoku**	1 (5.6)	1 (2.1)		4 (9.3)	6 (5.7)	
**Kanto**	4 (22.2)	8 (16.7)		9 (11.6)	22 (20.8)	
**Chubu**	1 (5.6)	19 (39.6)		5 (20.9)	19 (17.9)	
**Kinki**	8 (44.4)	7 (14.6)		10 (23.3)	33 (31.1)	
**Chugoku**	0	5 (10.4)		4 (9.3)	6 (5.7)	
**Shikoku**	0	1 (2.1)		2 (4.7)	1 (0.9)	
**Kyushu-Okinawa**	4 (22.2)	3 (6.2)		8 (18.6)	13 (12.3)	
**Hospital type**						
**National hospital**	2 (11.1)	5 (10.4)	0.88	4(9.5)	10(9.5)	0.14
**Public medical hospital**	9 (50.0)	19 (39.6)		16(38.1)	47(44.8)	
**Social insurance hospital**	0 (0.0)	4 (8.3)		3(7.1)	0(0.0)	
**Healthcare corporation**	3 (16.7)	9 (18.8)		9(21.4)	25(23.8)	
**Private clinic and others**	4 (22.2)	11 (22.9)		10(23.8)	23(21.9)	
**No response**				1	1	
**Number of Beds**						
**Beds < 400**	13 (72.2)	27 (56.2)	0.27	10 (23.3)	32 (30.2)	0.42
**Beds ≥ 400**	5 (27.8)	21 (43.8)		33 (76.7)	74 (69.8)	
**Internal medicine or Surgery**						
**Internal medicine**	12 (66.7)	32 (66.7)	>0.99	25 (58.1)	75 (70.8)	0.22
**Surgery**	1 (5.6)	4 (8.3)		7 (16.3)	9 (8.5)	
**Mixed**	5 (27.8)	12 (25.0)		11 (25.6)	22 (20.8)	
**Enrollment of CN or CNS**						
**Yes**	14 (77.8)	31 (64.6)	0.38	21 (48.8)	40 (37.7)	0.27
**No**	4 (22.2)	17 (35.4)		22 (51.2)	66 (62.3)	
**Enrollment of the hepatitis medical care coordinator**						
**Yes**	10 (55.6)	26 (54.2)	>0.99	16 (37.2)	28 (26.4)	0.24
**No**	8 (44.4)	22 (45.8)		27 (62.8)	78 (73.6)	
**Workshops or trainings about sarcopenia**						
**Yes**	7 (38.9)	11 (22.9)	0.22	14 (32.6)	16 (15.1)	0.02
**No**	11 (61.1)	37 (77.1)		29 (67.4)	90 (84.9)	
**Use of sarcopenia diagnostic criteria**						
**Yes**	6 (33.3)	1 (2.1)	<0.01	18 (41.9)	4 (3.8)	<0.01
**No**	12 (66.7)	47 (97.9)		25 (58.1)	102 (96.2)	
**Enrollment of the ward-specific physiotherapist**						
**Yes**				18 (41.9)	69 (65.1)	0.01
**No**				25 (58.1)	37 (34.9)	
**Enrollment of the ward-specific dietitian**						
**Yes**				33 (76.7)	82 (77.4)	>0.99
**No**				10 (23.3)	24 (22.6)	

Results are presented as number (%). Location was expressed by grouping prefectures into regions. EG, evaluation group; NEG, non-evaluation group; CN, certified nurse; CNS, certified nurse specialist.

**Table 5:** Comparison between intervention group and non-intervention group in outpatient department and ward.

	Outpatient Department			Ward		
	IG (*n* = 13)	NIG (*n* = 53)	*p*-Value	IG (*n* = 29)	NIG (*n* = 120)	*p*-Value
**Location**						
**Hokkaido**	2 (15.4)	2 (3.8)	0.64	1 (3.4)	6 (5.0)	0.28
**Tohoku**	1 (7.7)	1 (1.9)		3 (10.3)	7 (5.8)	
**Kanto**	2 (15.4)	10 (18.9)		2 (6.9)	29 (24.2)	
**Chubu**	4 (30.8)	16 (30.2)		8 (27.6)	16 (13.3)	
**Kinki**	3 (23.1)	12 (22.6)		8 (27.6)	35 (29.2)	
**Chugoku**	0	5 (9.4)		2 (6.9)	8 (6.7)	
**Shikoku**	0	1 (1.9)		0	3 (2.5)	
**Kyushu-Okinawa**	1 (7.7)	6 (11.3)		5 (17.2)	16 (13.3)	
**Hospital type**						
**National hospital**	1 (7.7)	6 (11.3)	>0.99	3 (10.7)	11 (9.2)	0.91
**Public medical hospital**	6 (46.2)	22 (41.5)		14 (50.0)	49 (41.2)	
**Social insurance hospital**	1 (7.7)	3 (5.7)		0	3 (2.5)	
**Healthcare corporation**	2 (15.4)	10 (18.9)		5 (17.9)	29 (24.4)	
**Private clinic and others**	3 (23.1)	12 (22.6)		6 (21.4)	27 (22.7)	
**No response**				1	1	
**Number of Beds**						
**Beds < 400**	4 (30.8)	22 (41.5)	0.54	4 (13.8)	38 (31.7)	0.07
**Beds ≥ 400**	9 (69.2)	31 (58.5)		25 (86.2)	82 (68.3)	
**Internal medicine or Surgery**						
**Internal medicine**	10 (76.9)	34 (64.2)	0.78	20 (69.0)	80 (66.7)	>0.99
**Surgery**	0	5 (9.4)		3 (10.3)	13 (10.8)	
**Mixed**	3 (23.1)	14 (26.4)		6 (20.7)	27 (22.5)	
**Enrollment of CN or CNS**						
**Yes**	35 (66.0)	10 (76.9)	0.53	14 (48.3)	47 (39.2)	0.41
**No**	18 (34.0)	3 (23.1)		15 (51.7)	73 (60.8)	
**Enrollment of the hepatitis medical care coordinator**						
**Yes**	5 (38.5)	25 (47.2)	0.76	10 (34.5)	34 (28.3)	0.51
**No**	8 (61.5)	28 (52.8)		19 (65.5)	86 (71.7)	
**Workshops or training about sarcopenia**						
**Yes**	11 (84.6)	7 (13.2)	<0.01	12 (41.4)	18 (15.0)	<0.01
**No**	2 (15.4)	46 (86.8)		17 (58.6)	102 (85.0)	
**Nutrition Support Team**						
**Yes**	12 (92.3)	52 (98.1)	0.36	29	120	
**No**	1 (7.7)	1 (1.9)				
**Use of sarcopenia diagnostic criteria**						
**Yes**	3 (23.1)	4 (7.5)	0.13	13 (44.8)	9 (7.5)	<0.01
**No**	10 (76.9)	49 (92.5)		16 (55.2)	111 (92.5)	
**Enrollment of the ward-specific physiotherapist**						
**Yes**				18 (62.1)	69 (57.5)	0.68
**No**				11 (37.9)	51 (42.5)	
**Enrollment of the ward-specific dietitian**						
**Yes**				24 (82.8)	91 (75.8)	0.62
**No**				5 (17.2)	29 (24.2)	

Results are presented as number (%). Location was expressed by grouping prefectures into regions. IG, intervention group; NIG, non-intervention group; CN, certified nurse; CNS, certified nurse specialist.

**Table 6:** Details regarding prevention or treatment of sarcopenia as determined using open-ended questions.

	Category (n)	Subcategory
Outpatient Department	Exercise (2)	∙ Exercise therapy for preventing sarcopenia provided by a physiotherapist
	Nutrition (2)	∙ Nutritional guidance provided by a dietician
	Mixed (4)	∙ Rehabilitation and dietary supplementation ∙ Nutritional guidance and education regarding exercises for preventing sarcopenia
	Others (4)	∙ Patient education regarding sarcopenia provided by healthcare professionals ∙ Follow up for outpatients with liver disease by a medical team (including doctor, nurse, dietician, and pharmacist)
Ward	Exercise (6)	∙ Education regarding ways to exercise to sarcopenia provided in the form of a health brochure ∙ Exercise guidance provided by a nurse ∙ Exercise therapy provided by a physiotherapist
	Nutrition (7)	∙ Nutritional consultation or nutritional guidance provided by a dietician ∙ Providing information regarding sarcopenia
	Mixed (11)	∙ Nutritional guidance and exercise therapy ∙ Nutritional therapy and exercise therapy ∙ Providing information regarding exercise and a healthy diet ∙ Promoting walking and providing nutritional and medication guidance
	Others (1)	∙ Assessing walking ability and activities of daily living at the time of admission and discharge ∙ Assessing the necessity of nutritional support once per week

Mixed includes exercise and nutrition.

## Data Availability

The data presented in this study are available on request from the corresponding author.
